# The Siconolfi step test: a valid and reliable assessment of cardiopulmonary fitness in older men with prostate cancer

**DOI:** 10.1186/s11556-018-0207-9

**Published:** 2019-01-10

**Authors:** Agnieszka Lemanska, Karen Poole, Jonathan J. Aning, Bruce A. Griffin, Ralph Manders, John M. Saxton, Joe Wainwright, Sara Faithfull

**Affiliations:** 1 Faculty of Health and Medical Sciences, University of Surrey, Guildford, UK; 2 Department of Urology, Freeman Hospital, Newcastle Upon Tyne Hospitals NHS Foundation Trust, Newcastle Upon Tyne, UK; 3 Bristol Urological Institute, Southmead Hospital, Westbury-on-trym, Bristol, UK; 4 Department of Sport, Exercise & Rehabilitation, Northumbria University, Newcastle Upon Tyne, UK

**Keywords:** Siconolfi step test, Cardiopulmonary fitness, Prostate cancer, Prehabilitation, Clinical assessment

## Abstract

**Background:**

Assessing fitness and promoting regular physical activity can improve health outcomes and early recovery in prostate cancer. This is however, underutilised in clinical practice. The cardiopulmonary exercise test (CPET) is increasingly being used pre-treatment to measure aerobic capacity and peak oxygen consumption (VO_2_peak - a gold standard in cardiopulmonary fitness assessment). However, CPET requires expensive equipment and may not always be appropriate. The Siconolfi step test (SST) is simpler and cheaper, and could provide an alternative.

The aim of this study was to evaluate the validity and reliability of SST for predicting cardiopulmonary fitness in men with prostate cancer. Men were recruited to this two-centre study (Surrey and Newcastle, United Kingdom) after treatment for locally advanced prostate cancer. They had one or more of three risk factors: elevated blood pressure, overweight (BMI > 25), or androgen deprivation therapy (ADT). Cardiopulmonary fitness was measured using SST and cycle ergometry CPET, at two visits three months apart. The validity of SST was assessed by comparing it to CPET. The VO_2_peak predicted from SST was compared to the VO_2_peak directly measured with CPET. The reliability of SST was assessed by comparing repeated measures. Bland-Altman analysis was used to derive limits of agreement in validity and reliability analysis.

**Results:**

Sixty-six men provided data for both SST and CPET. These data were used for validity analysis. 56 men provided SST data on both visits. These data were used for reliability analysis. SST provided valid prediction of the cardiopulmonary fitness in men > 60 years old. The average difference between CPET and SST was 0.64 ml/kg/min with non-significant positive bias towards CPET (*P* = 0.217). Bland-Altman 95% limits of agreement of SST with CPET were ± 7.62 ml/kg/min. SST was reliable across the whole age range. Predicted VO_2_peak was on average 0.53 ml/kg/min higher at Visit 2 than at Visit 1 (*P* = 0.181). Bland-Altman 95% limits of agreement between repeated SST measures were ± 5.84 ml/kg/min.

**Conclusions:**

SST provides a valid and reliable alternative to CPET for the assessment of cardiopulmonary fitness in older men with prostate cancer. Caution is advised when assessing men 60 years old or younger because the VO_2_peak predicted with SST was significantly lower than that measured with CPET.

## Introduction

Prostate cancer is the most common cancer in UK men with almost 47,000 men diagnosed every year [1]. Higher prevalence is associated with older age and 54% of all new cases are in men aged 70 and over [1]. Poorer survival is also associated with older age, in particular for people over 70 [2]. The reasons for this include multi-morbidity, which can make older men less able to tolerate treatment and its adverse effects resulting in worse adherence and non-completion [1, 2]. Furthermore, poor levels of fitness may impact negatively on clinician and patient decision-making and consequently reduce access to curative cancer treatment [3, 4]. With the advent of early chemotherapy for advanced disease and more complex adjuvant therapies, assessment of fitness prior to prostate cancer treatment is needed to optimise clinical outcomes in older men.

Cardiopulmonary fitness has traditionally been assessed before radical prostatectomy, as part of enhanced recovery pathways [5] or prior to prehabilitation [6]. It is well recognised that surgery can have a significant impact on catabolism and oxygen demand, and the length and extent of surgery is directly related to the risk of developing post-surgery complications [7]. Eligibility for radical prostatectomy is often based on chronological age, but evidence shows that post-surgery complications are affected more by comorbidities than age [8]. In clinical trials, even if there is no age limit, people are required to be “fit for treatment” as drug tolerance may decrease and toxicity may increase in those with poor fitness [9]. For example, in the STAMPEDE prostate cancer trial, only men without a history of significant cardiovascular disease were recruited, reducing the number of older men in the study [10]. Comorbidities can therefore be a significant barrier to clinical trial entry [3].

Assessing patients in order to decide on the most appropriate treatment can be complex. Integrating functional and specialist assessments has been proposed as part of the international guidance for managing prostate cancer [11]. The health status of older people with cancer can be very diverse. This means that they need a tailored approach to treatment that considers their cardiopulmonary fitness as well as their functional performance status [12]. Cardiopulmonary fitness is associated with cardiovascular risk [13, 14], and like physical strength, can be improved by physical activity [15, 16].

People with cancer in comparison to age-matched people without cancer, have lower levels of cardiopulmonary fitness [17–19]. This can be attributed, in part, to treatment morbidity, but may also be linked to a sedentary lifestyle, which has been found to increase the risks of some cancers, including prostate cancer [20–23]. Exercise has been shown to alleviate Androgen Deprivation Therapy (ADT) related symptoms [24] and increase survival in prostate cancer [25, 26]. However, the design and delivery of appropriate lifestyle interventions requires a safe and simple assessment of fitness, to provide a personalised exercise prescription as a part of prehabilitation and rehabilitation programmes. Furthermore, it can provide an important pre-treatment benchmark to motivate patients to improve or sustain their physical activity levels. The development of a cheap and easy to implement assessment that provides a valid and reliable measure of fitness remains a challenge.

The cardiopulmonary exercise test (CPET) provides a direct measurement of aerobic capacity and peak oxygen consumption (VO_2_peak) and is a gold standard in preoperative cardiopulmonary fitness assessment [27, 28]. In prostate cancer, CPET is used for physical assessment and to evaluate preoperative risks [29], alongside the American Society of Anesthesiologists Physical Status Classification System [30, 31]. However, wider implementation of CPET in routine clinical practice is limited due to unavailability or high costs. The Siconolfi step test (SST) is simpler (it can be performed in any clinical or non-clinical setting) and cheaper than CPET. It has been validated to predict VO_2_peak in healthy adults [32, 33]. It has also been evaluated as a cardiopulmonary fitness assessment in patients with systemic lupus erythematosus [34] and rheumatoid arthritis [35]. To our knowledge, there is no study to evaluate the validity and reliability of SST in men with prostate cancer. SST is performed at a submaximal intensity of exercise, making it safe and suitable for elderly and frail men or people with disability. In this study, we evaluated SST against CPET and reported on the validity and reliability of SST in predicting cardiopulmonary fitness in men with prostate cancer. The reliability refers to the stability of SST in predicting cardiopulmonary fitness across time.

## Methods

### Study design

Patients were recruited from two centres: the Royal Surrey County Hospital NHS Foundation Trust, and the Newcastle upon Tyne Hospitals NHS Foundation Trust. SST and CPET were first performed at Visit 1 and then repeated at Visit 2 three months later. Participants were given their SST and CPET assessment results, but no lifestyle advice or health intervention was provided.

### Study population

Men with localised prostate cancer at 3–36 months’ post-diagnosis and with stable PSA (< 0.4 ng/ml surgery and radiotherapy patients, and < 10 ng/ml androgen deprivation therapy (ADT) patients) were invited to participate. They were recruited only if they had one or more of the three risk factors: BMI < 18.5 or > 25; elevated blood pressure; receiving ADT. To enter the study, men would have completed their prostate cancer treatment a minimum of 3 months before (6 months for brachytherapy). Men with a history of cardiovascular or pulmonary disease or receiving active cancer treatment (apart from ADT) were excluded.

### Dataset

**Fig. 1:**
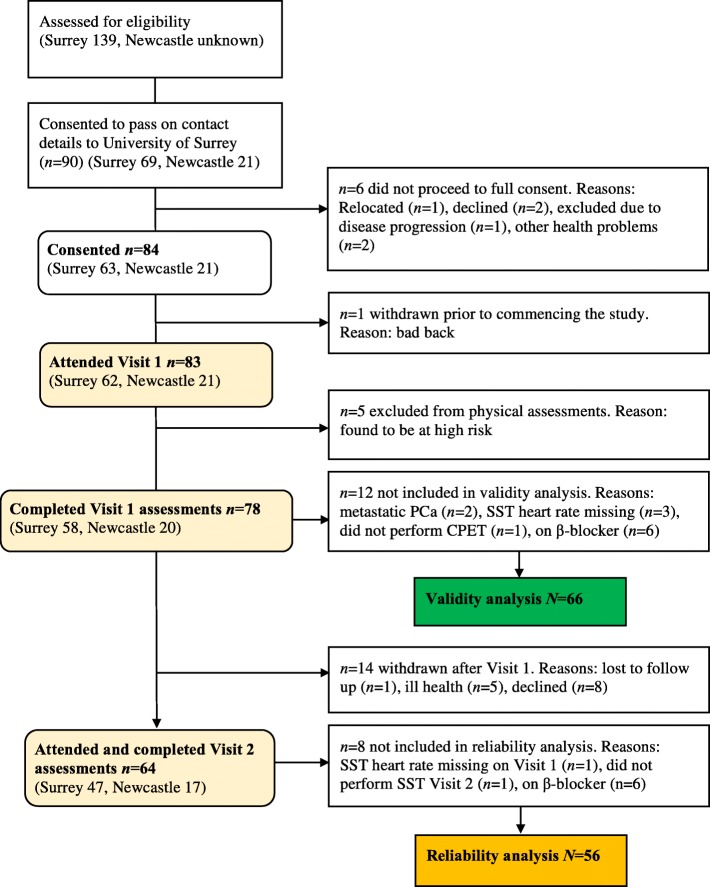
Study participants CONSORT diagram

A total of 83 men participated in the study at two centres, and 64 attended both visits (23% attrition). The study consort diagram is presented in Fig. 1. Data for 66 participants who provided both SST and CPET data at Visit 1 were available for SST validity analysis. Out of the 83 study participants, five were excluded from Visit 1 physical assessments (both SST and CPET) during the medical check, due to risks identified in the screening Physical Activity Readiness Questionnaire [36]. An additional 12 participants were not included in validity analysis because they had metastatic cancer, missing SST or CPET data, or were taking beta-adrenergic blocking medications (inhibition of heart rate elevation during exercise).

Data for 56 participants who delivered SST data at both Visits 1 and 2 were available for SST reliability analysis. Out of the 64 men who attended both visits, eight were excluded from reliability analysis due to missing SST data or taking beta-adrenergic blocking medications. Demographic and treatment characteristics, Charlson Comorbidity Index (CCI) [37], hand grip strength [38, 39] and chair sit to stand test [40] results in the total population, and validity and reliability cohorts are shown in Table 1.

**Table 1:** Demographic and treatment characteristics of the study population & validity and reliability cohorts

	Study population *N* = 83	Validity cohort *N* = 66	Reliability cohort *N* = 56	*P* value
Centre				1.000
Surrey: *n* (%)	62 (74.7)	52 (78.8)	44 (78.6)	
Newcastle: *n* (%)	21 (25.3)	14 (21.2)	12 (21.4)	
Age (years)				0.943
mean (SD)	68.2 (7.4)	68.1 (7.0)	68.2 (7.3)	
range	47–83	47–83	47–83	
≤ 60 *n* (%)	14 (16.9)	10 (15.2)	9 (16.1)	
> 60 *n* (%)	69 (83.1)	56 (84.8)	47 (83.9)	
Ethnicity				0.199
White: *n* (%)	80 (96.4)	63 (95.4)	53 (94.6)	
Black Caribbean: *n* (%)	2 (2.4)	2 (3.0)	2 (3.6)	
Black African: *n* (%)	1 (1.2)	1 (1.5)	1 (1.8)	
Treatment (men had combined treatments)				0.996
Surgery: *n* (%)	53 (63.9)	43 (65.1)	36 (64.3)	
Radiotherapy: *n* (%)	26 (31.3)	23 (34.8)	19 (33.9)	
Brachytherapy: *n* (%)	3 (3.6)	3 (4.5)	3 (5.3)	
ADT: *n* (%)	32 (38.6)	26 (39.4)	21 (37.5)	
Smoking status				0.926
Non smoker: *n* (%)	45 (54.2)	36 (54.5)	32 (57.1)	
Ex-smoker: *n* (%)	28 (33.7)	23 (34.8)	19 (33.9)	
Smoker: *n* (%)	3 (3.6)	3 (4.5)	3 (5.4)	
Missing: *n* (%)	7 (8.4)	4 (6.1)	2 (3.6)	
Retirement				0.970
Yes: *n* (%)	19 (22.9)	16 (24.2)	14 (25.0)	
No: *n* (%)	54 (65.1)	42 (63.6)	36 (64.3)	
Missing: *n* (%)	10 (12.0)	8 (12.1)	6 (10.7)	
Height (cm): mean (SD)	175.5 (6.5)	175.9 (6.5)	175.5 (6.6)	0.713
Weight (kg): mean (SD)	89.2 (11.8)	88.6 (11.6)	88.7 (11.2)	0.980
Diabetes: *n* (%)	7 (8.4)	4 (6.1)	3 (5.3)	1.000
Resting blood pressure, systolic (mmHg): mean (SD)	135.7 (15.3)	135.3 (15.1)	136.5 (15.1)	0.651
Resting blood pressure, diastolic (mmHg): mean (SD)	81.4 (10.0)	80.7 (9.1)	81.0 (9.2)	0.853
Waist circumference (cm): mean (SD)	102.2 (9.7)	101.4 (9.4)	101.7 (9.0)	0.886
Hip circumference (cm): mean (SD)	105.5 (7.6)	104.7 (7.3)	104.8 (6.8)	0.921
Grip strength (kg): mean (SD)	38.4 (8.4)	39.3 (7.6)	39.2 (7.8)	0.934
Chair sit to stand (number completed): median (IQR)	13 (5)	13 (5)	13 (5)	0.608
Charlson co-morbidity index (CCI): median (IQR)	6 (3)	5 (2.5)	6 (3)	0.848

Column 1 describes all study participants. Column 2 and 3 describe the cohorts used for Validity (*N* = 66) and Reliability analysis (*N* = 56). *P* value was calculated to statistically assess the difference between the validity and reliability cohorts

### Fitness assessments

#### Cardiopulmonary exercise test (CPET)

The participants completed a continuous, incremental exercise test to volitional exhaustion on an electronically braked cycle ergometer. The pedalling frequency was self-selected within a range of 60–90 rpm. After a two minute warm up against no resistance (0 watt), the intensity of exercise was increased by 20–30 watts/minute. Participants were encouraged to continue cycling to volitional exhaustion or until a plateau in oxygen consumption was observed. Heart rate (HR) and volume of oxygen (V0_2_) consumed during exercise (ml/kg/min) were measured. Peak oxygen consumption (VO_2_peak) was calculated as the highest consecutive 20 s period of gas exchange data in the last minute before volitional exhaustion. Participants were not trained athletes and so unaccustomed to maximum physical exertion. Therefore, a submaximal test was performed, terminated at volitional exhaustion and VO_2_peak (not VO_2_max) was recorded [41, 42].

#### Siconolfi step test (SST)

Participants were required to step up and down from a portable 10-in. (25.4 cm) step for a maximum of three, three minute stages. There was one minute rest between stages. The stepping cadence, timed using a metronome, was increased at each stage. Stage 1 was at a rate of 17 steps per minute, stage 2 at 26 steps per minute and stage 3 at 34 steps per minute [33]. HR was monitored during and after each stage.

### Statistical analysis

VO_2_peak for each patient was directly measured with CPET and also predicted from SST according to equations by Siconolfi et al. [33, 43]. The validity of SST was assessed against CPET from the data collected at Visit 1. The reliability of SST was based on two repeated measures from Visit 1 and Visit 2. The Pearson correlation coefficient (r) was used to explore the strength and significance of the relationship between SST and CPET (validity analysis), and between repeated SST measures (reliability analysis). The Intraclass correlation coefficient (ICC) and paired t-test were used to measure and test the agreement between SST and CPET, and between repeated SST measures. Finally the Bland-Altman method [44] was used to calculate the 95% limits of agreement (LOA) between CPET and STT, and between repeated SST measures. The smaller the range between the lower and upper LOA in validity analysis, the greater the validity of SST is in predicting VO_2_peak measured with CPET. Univariate and multivariate linear regression was used to examine factors that contribute to the difference between SST and CPET. Factors such as age, BMI, CCI, grip strength, and chair sit to stand test results were included in the regression analysis. Statistical significance was considered at *P* < 0.05. The dataset was entered and managed in SPSS version 22 (SPSS Inc., Chicago, USA). Statistical analyses were performed in R version 3.0.2 (R Development Core Team, Austria).

### Missing data

Data on body weight was missing from one participant at Visit 2. This was imputed using single regression imputation [45]. SST or CPET missing data were not imputed and only complete cases for Visit 1 and Visit 2 were included in validity and reliability analysis. This resulted in a smaller sample available for the reliability analysis than for the validity analysis. However, to preserve the maximum available sample number, a different number of samples was used for each of the analyses.

## Results

### Sample characteristics

Sample demographic characteristics and treatment data are presented in Table 1. In total, 66 men (52 from Surrey and 14 from Newcastle) with a mean age 68.1 ± 7.0 years were included in the validity analysis and 56 men (mean age 68.2 ± 7.3 years) who provided both Visit 1 and Visit 2 SST data, were included in the reliability analysis. There was no statistically significant difference in patient characteristics between the validity and reliability cohorts (Table 1). Most men recruited to the study (96.4%) were of white ethnicity and only 3 men (3.6%) were of black ethnicity. Most men did not smoke (87.9%), with only 3 (3.6%) reporting that they smoked during the study. 65.1% of men were still employed. 63.9% were treated with surgery, 31.3% with radiotherapy and 38.6% of men had ADT.

### SST validity analysis

Table 2 shows the results of the Visit 1 SST and CPET for the validity cohort (*N* = 66), and for the subsample of patients > 60 years old (*n* = 56). For the whole validity cohort, the average VO_2_peak predicted from SST was 19.5 ± 5.1 ml/kg/min and HR at completion was 141.6 ± 20.7 beats per minute (bpm). The VO_2_peak measured with CPET was on average 1.61 ± 4.71 ml/kg/min higher than that predicted with SST. This was statistically significant (*P* = 0.010). The correlation between predicted and measured VO_2_peak was *r* = 0.69 (*p* < 0.001, 95%CI 0.54 to 0.80) and ICC was 0.64 (*p* < 0.001, 95%CI 0.48 to 0.77).

**Table 2:** Validity analysis of Siconolfi step test (SST) against cardiopulmonary exercise test (CPET)

Validity analysis	Validity cohort (*N* = 66)	> 60 years olds (*n* = 56)
SST		
Predicted VO_2_peak (ml/kg/min):		
average (SD)	19.5 (5.1)	19.2 (5.2)
range	10.8–35.5	10.8–35.5
Heart rate (bpm): mean (SD)	141.6 (20.7)	142.2 (21.2)
CPET		
Measured VO_2_peak (ml/kg/min):		
average (SD)	21.1 (6.5)	19.8 (5.1)
range	9.9–40.2	9.9–32.5
Heart rate (bpm): mean (SD)	148.7 (15.9)	147.5 (16.2)
Pearson correlation		
r	0.69	0.73
*P* value	< 0.001	< 0.001
0.95% CI	0.54–0.80	0.58–0.83
Paired t-test		
average difference	1.61	0.64
*P* value	0.010	0.217
0.95% CI	0.43–3.09	-0.38–1.66
ICC		
ICC	0.64	0.73
*P* value	< 0.001	< 0.001
0.95% CI	0.48–0.77	0.58–0.83

Results are presented for all patients that participated in Visit 1 and provided valid SST and CPET data (validity cohort, *N* = 66) (column 1), and the subsample of patients older than 60 years (*n* = 56) (column 2). Validity is assessed with Pearson correlation (r), paired t-test and intraclass correlation coefficient (ICC). Statistical significance was considered at level of *P* < 0.05

### The effect of age on validity of SST: Regression analysis

**Fig. 2:**
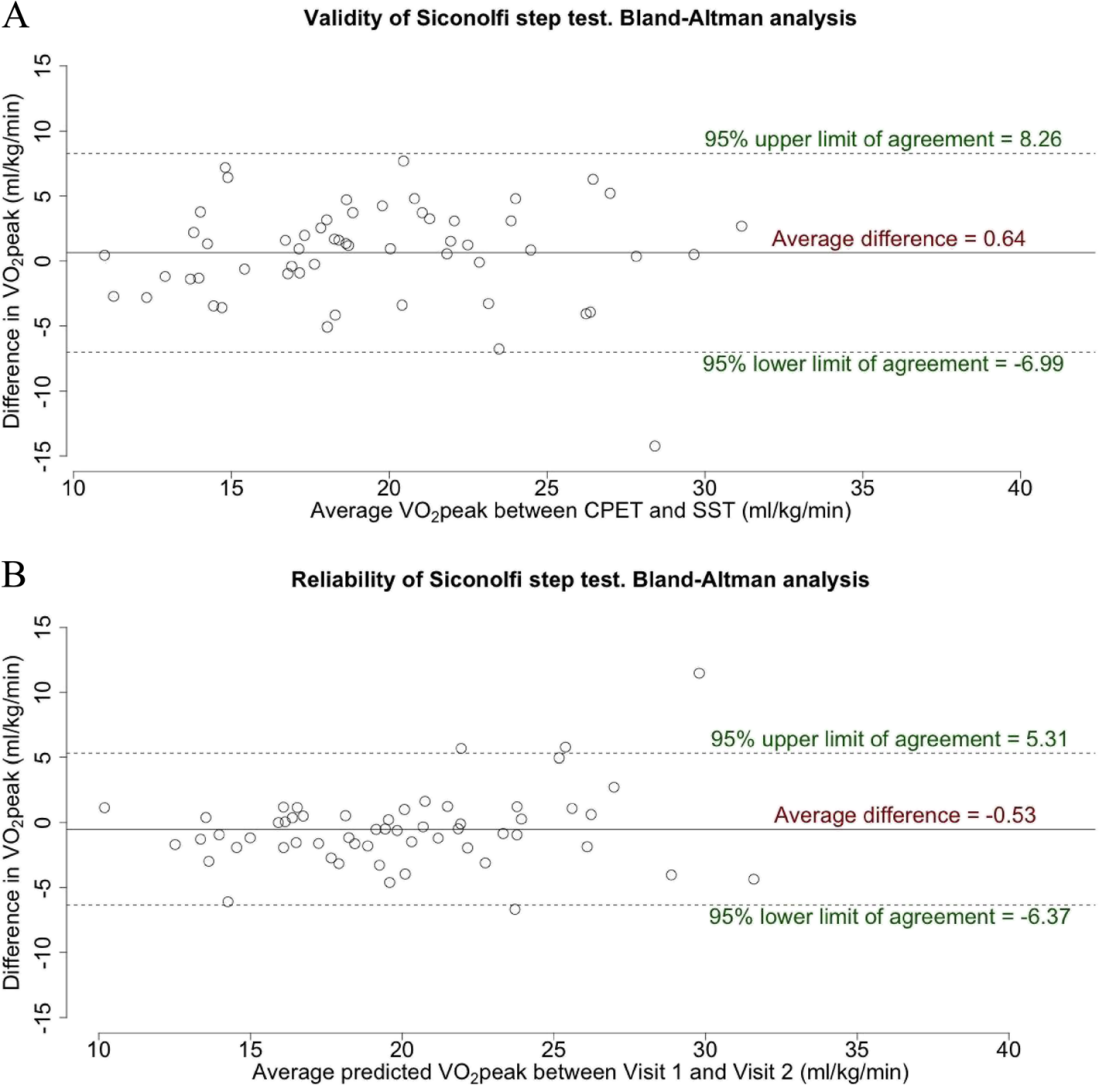
Bland-Altman analysis. **a**) Validity of Siconolfi step test (SST) against cardiopulmonary exercise test (CPET) using data for *n* = 56 participants > 60 years old (≤ 60 years olds were removed according to the results of regression analysis). **b**) Reliability of Siconolfi step test (SST) using data for *N* = 56 participants that provided valid SST data at both visits

Age made a significant contribution to the difference between SST and CPET, being responsible for 16% of variance. The univariate regression coefficient was − 0.27 (*p* < 0.001) and the multivariate regression coefficient was − 0.23 (*p* = 0.009), which indicates that the difference was greater for younger men. Other factors included in the multivariate model (BMI, CCI, grip strength and sit to stand test) were not statistically significant. When men 60 years old or younger (*n* = 10) were removed from the validity analysis the average difference between SST and CPET decreased to 0.64 ± 3.81 ml/kg/min, which was not statistically significant (*p* = 0.217). The LOA between SST and CPET from Bland-Altman validity analysis (Fig. 2a), were ± 7.62 ml/kg/min.

### SST reliability analysis

Table 3 shows the results of SST and CPET at Visit 1 and Visit 2. The average VO_2_peak values predicted from SST for the reliability cohort (*N* = 56) were 19.6 ± 5.2 ml/kg/min at Visit 1 and 20.2 ± 4.5 ml/kg/min at Visit 2. The difference of 0.53 ml/kg/min was not statistically significant (*P* = 0.181). Pearson correlation coefficient *r* between visits was 0.83 (*p* < 0.001, 95% CI 0.72 to 0.89) and ICC was 0.81 (*p* < 0.001, 95% CI 0.70 to 0.89). The 95% Bland-Altman LOA (Fig. 2b) were from ±5.84 ml/kg/min.

**Table 3:** Reliability analysis of Siconolfi step test (SST)

Reliability analysis	Visit 1 (*N* = 56)	Visit 2 (*N* = 56)
SST		
Predicted VO_2_peak (ml/kg/min):		
average (SD)	19.6 (5.2)	20.2 (4.5)
range	10.8–35.5	9.6–33.8
Heart rate (bpm): mean (SD)	142.2 (21.3)	142.9 (20.3)
Pearson correlation		
r	0.83	
*P* value	< 0.001	
0.95% CI	0.72–0.89	
Paired t-test		
average difference	0.53	
*P* value	0.181	
0.95% CI	-0.25–1.31	
ICC		
ICC	0.81	
*P* value	< 0.001	
0.95% CI	0.70–0.89	

Repeated measures from Visit 1 (column 1) and Visit 2 (column 2) are presented for *N* = 56 participants that provided valid SST data at both visits. Reliability is assessed with Pearson correlation (r), paired t-test and intraclass correlation coefficient (ICC). Statistical significance was assessed at level of *P* < 0.05

### The effect of age on validity and reliability of SST – Comparison of results from visit 1 and visit 2

**Fig. 3:**
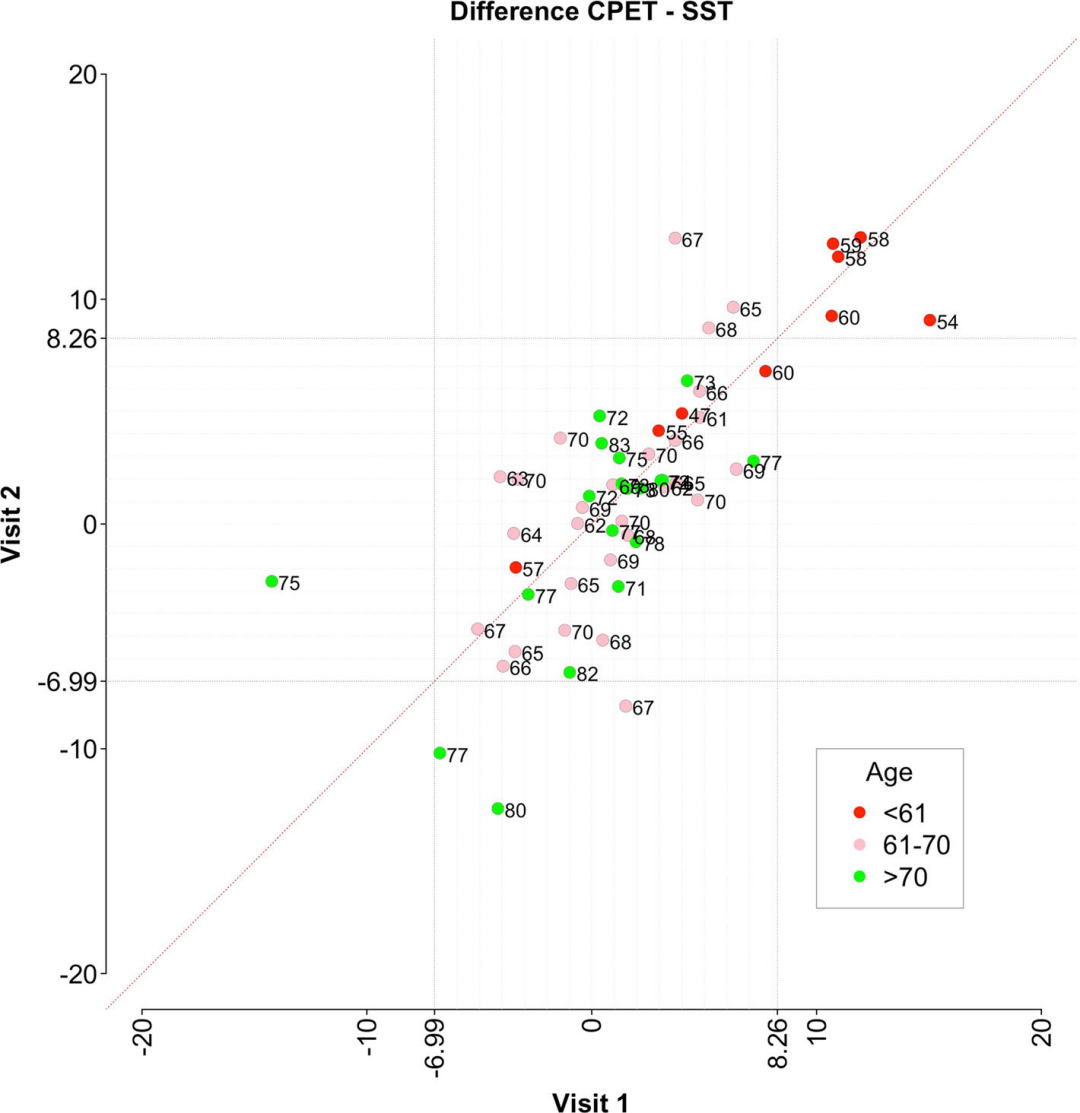
Scatter plot of differences between cardiopulmonary exercise test (CPET) and Siconolfi step test (SST) at Visit 1 vs Visit 2. The numbers next to markers show the age of participants. Men ≤ 60 years old are marked in red. The diagonal line is y = x. Vertical and horizontal lines represent Bland-Altman limits of agreement (LOA) (Fig. 2a)

Figure 3 illustrates the distribution of differences between SST and CPET for Visit 1 against Visit 2. SST was shown to be highly reliable across the entire age range, as indicated by values falling along and very close to the diagonal (y = x) line. However, most values for men of 60 years and younger are outside the upper Bland-Altman LOA on both visits. This confirms the results of the regression analysis and indicates poor validity of SST for men ≤ 60 years old. CPET VO_2_peak values for these men were significantly and consistently higher than those predicted with SST at both visits.

### Acceptability of SST and adverse events

SST was found to be simple to perform and was well tolerated, with no adverse events. The test was acceptable to participants with a limited capacity to perform physical exercise. However, in respect of the latter, caution is advised when recommending SST to men with comorbidities that limit their ability to exercise or compromise their balance and lower limb function. In addition, men on beta-blocking medications that inhibit HR elevation during exercise and render the prediction of VO_2_peak invalid, should also be excluded.

## Discussion

The purpose of this study was to propose a valid and reliable methodology for a rapid fitness evaluation that requires minimal space and was easy to implement. Step tests have traditionally been used as an alternative to CPET for assessing cardiopulmonary fitness (e.g. Chester Step Test) [46–49]. SST was selected because it has previously been validated to predict VO_2_peak in healthy adults [32, 33] and in other clinical populations [34, 35]. The benefits of SST over CPET include simpler and cheaper implementation as well as equipment that is easily obtainable and transportable. An important advantage of SST is also that it fits within limited space e.g. a small clinical room and it can be delivered by non-clinical staff with minimal training. In this context, SST can also be safer than CPET because it is performed at a submaximal intensity of exercise.

Notably SST is one of many tests developed for a safe and pragmatic assessment of cardiopulmonary fitness. Other submaximal testing procedures that can be used for indirect estimation of VO_2_peak include modified exercise ergometry protocols without spirometry, such as the Astrand-Ryhming nomogram to predict cardiopulmonary fitness [43]. However, the main limitation of these assessments is that they require an expensive research-grade cycle ergometer. Treadmill protocols also exist, as well as walking or running tests [50, 51], but they are not pragmatic in the context of non-laboratory, minimal space settings.

This study identifies the important issue of the need for evaluation of cardiopulmonary fitness of men with prostate cancer. The assessment is not only important in risk stratification of men prior to treatment. It also can be used to guide personalised exercise recommendations to optimise prehabilitation and rehabilitation interventions. The benefits of exercise on health-related quality of life are well evidenced in systematic reviews of randomized controlled trials [52, 53]. Improving fitness has been shown to improve health outcomes and reduce cancer-related symptoms [54, 55]. Furthermore, increased physical activity is associated with reduced risk of recurrence and improved survival [26].

The aim of this study was to provide a scalable solution to these problems by improving men’s engagement in physical activity and their fitness. A growing body of evidence in support of the benefits of physical activity has led to the publication of exercise guidelines for cancer survivors [56, 57]. Despite this, it is estimated that only 10–32% of cancer survivors meet the recommended physical activity levels [58]. Therefore, an important first step in promoting physical activity, is to define individual fitness levels. The results of this study show that SST can provide a pragmatic and scalable alternative to CPET for the assessment of cardiopulmonary fitness in men with prostate cancer. It can be used to help tailor physical activity interventions to the needs and priorities of individual patients.

Barriers to the routine implementation of a fitness assessment in men with prostate cancer include resource constraints, time pressures to begin treatment and limited evidence regarding the benefits of testing men prior to treatment. CPET is expensive as it requires highly trained staff, specialist facilities and equipment such as a stationary exercise bike or a treadmill as well as ECG and oxygen uptake monitoring systems. While the British Thoracic Society 2017/2018 guidance [56] states that CPET in outpatient or day-case settings in the UK costs £244, this does not cover the capital expenditure to purchase CPET equipment. In comparison, SST requires significantly less resources, no specialist equipment and less space. The equipment (a 10 in. (25.4 cm) step and a heart rate monitor) is cheap (total capital expenditure is approximately £162), small and portable.

CPET is a well validated benchmark measure of cardiopulmonary fitness, and it was used here to validate SST. This is an important strength of this study. In addition, both CPET and SST were performed twice in the same population, with the repeated measures allowing the reliability of SST to be assessed. Another strength of this study is the relatively large sample size, and a wide age range of participants (47–83) that is representative of the UK prostate cancer population. The reliability analysis cohort was 15% smaller than that which was available for the validity analysis. This was due to study attrition and missing data which are the main limitations of this study. In addition, VO_2_peak (not VO_2_max) was measured at the point of volitional termination. This is a more common measure in clinical populations of patients who are not trained athletes and unaccustomed to maximal intensity exercise. However, VO_2_peak is a validated measure of cardiopulmonary fitness and similarly to VO_2_max it represents cardiac output, vascularisation and oxygen utilisation by muscles [41, 42].

## Conclusions

This study highlights the importance of conducting validity and reliability work for SST as a predictor of VO_2_peak in men with prostate cancer. Age had a clear effect on the validity of SST for predicting cardiopulmonary fitness. For men aged 60 years and younger, SST predicted VO_2_peak values that were significantly lower than those measured with CPET. Caution is therefore advised when using SST to predict VO_2_peak in patients ≤ 60 years old, and further work is needed to establish the effect of age and HR on the validity of SST. Nevertheless, SST was a stable and reliable measure of fitness over time. In conclusion, these data present new evidence to support SST as a valid and reliable method for clinicians and rehabilitation specialists to assess and monitor cardiopulmonary fitness in men with prostate cancer. This assessment could be used to guide personalised exercise advice in pre- and post-treatment rehabilitation interventions. It could also be used in treatment decision making as it may help predict short and long-term outcomes of treatment. SST can be used in a wide range of clinical and non-clinical settings, and therefore could provide an alternative to the more expensive and resource demanding CPET.
